# Unconventional secretion of tau by VAMP8 impacts its intra- and extracellular cleavage

**DOI:** 10.3389/fcell.2022.912118

**Published:** 2022-10-05

**Authors:** Julie Pilliod, Maude Gélinas-Faucher, Nicole Leclerc

**Affiliations:** ^1^ Research Center of the University of Montreal Hospital (CRCHUM), Montréal, QC, Canada; ^2^ Département de Neurosciences, Faculté de Médecine, Université de Montréal, Montréal, QC, Canada

**Keywords:** tau protein, VAMP8, secretion, caspase-3, tau cleavage

## Abstract

In Alzheimer’s disease, Tau, a microtubule-associated protein, becomes hyperphosphorylated, detaches from microtubules, and accumulates in the somato-dendritic compartment where it forms insoluble aggregates. Tau also accumulates in the CSF of patients indicating that it is released by neurons. Consistent with this, several laboratories including ours have shown that Tau is secreted by neurons through unconventional secretory pathways. Recently, we reported that VAMP8, an R-SNARE found on late endosomes, increased Tau secretion and that secreted Tau was cleaved at the C-terminal. In the present study, we examined whether the increase of Tau secretion by VAMP8 affected its intra- and extracellular cleavage. Upon VAMP8 overexpression, an increase of Tau cleaved by caspase-3 in the cell lysate and medium was observed. This was correlated to an increase of active caspase-3 in the cell lysate and medium. Using a Tau mutant not cleavable by caspase-3, we demonstrated that Tau cleavage by caspase-3 was not necessary for its secretion upon VAMP8 overexpression. By adding recombinant Tau to the culture medium, we demonstrated that extracellular Tau cleavage by caspase-3 could occur because of the release of active caspase-3, which was the highest when VAMP8 was overexpressed. When cleavage of Tau by caspase-3 was prevented by using a non-cleavable mutant, secreted Tau was still cleaved at the C-terminal, the asparagine N410 contributing to it. Lastly, we demonstrated that N-terminal of Tau regulated the secretion pattern of a Tau fragment containing the microtubule-binding domain and the C-terminal of Tau upon VAMP8 overexpression. Collectively, the above observations indicate that VAMP8 overexpression affects the intra- and extracellular cleavage pattern of Tau.

## Introduction

Tau is a neuronal MAP enriched in the axon that becomes hyperphosphorylated, accumulates in the somato-dendritic compartment and self-aggregates into insoluble filaments called paired helical filaments (PHFs) forming the neurofibrillary tangles (NFTs) in Alzheimer’s disease (AD) (Ludin and Matus, 1993; Mandell & Banker, 1996; Lee et al., 2001; Cairns et al., 2007; Iqbal et al., 2016). Tau pathology is correlated to cognitive deficits in patients which, was confirmed by histopathological examination of post-mortem brain and Tau PET imaging (Tomlinson et al., 1970; Alafuzoff et al., 1987; Braak & Braak, 1991; Arriagada et al., 1992; Bierer et al., 1995; Ossenkoppele et al., 2016; Pontecorvo et al., 2019). The contribution of Tau dysfunction to neurodegeneration is further supported by the enrichment of Tau genetic variants in patients suffering from frontotemporal lobar degeneration (FTLD-Tau) (Cairns et al., 2007). No mutations in Tau gene were found in AD patients but Tau gene polymorphisms may be risk factors for sporadic AD (Schraen-Maschke et al., 2004). In a recent study, a duplication of the Tau gene was correlated to an early-onset dementia with an AD clinical phenotype (Le Guennec et al., 2017). Although all the above observations indicate that Tau pathology is involved in the pathogenesis of AD, its precise role in the process of neurodegeneration remains elusive.

Besides its intracellular accumulation, Tau also accumulates extracellularly in AD as revealed by its increase in the CSF during the progression of the disease. This increase was believed to correlate with neuronal cell death (Hampel et al., 2010). Several recent studies have demonstrated that Tau can be released by neurons through an active process of secretion (Pernegre et al., 2019). The presence of Tau in the interstitial fluid in the absence of neurodegeneration was detected by microdialysis in Tau transgenic mouse brain (Yamada et al., 2011). The release of Tau by neurons was shown to be increased by neuronal activity both *in vitro* and *in vivo* (Pooler et al., 2013; Yamada et al., 2014; Ismael et al., 2021). AD is linked to autophagic and lysosomal dysfunction, which was shown to increase the release of Tau by primary cortical neurons (Mohamed et al., 2014).

The secretory pathways of Tau are still largely unknown. So far, Tau was shown to be only secreted by unconventional pathways. Tau can be released either by its translocation across the plasma membrane or by membranous organelles that can fuse with the plasma membrane (Pernegre et al., 2019). Membranous organelles such as late endosomes, autophagosomes and lysosomes were shown to be involved in Tau release (Pernegre et al., 2019). In a previous study, we reported that Rab7A associated with late endosomes participates in Tau secretion (Rodriguez et al., 2017). More recently, we demonstrated that VAMP8, a R-SNARE associated with late endosomes, increases Tau secretion upon its overexpression in neurons and the neuronal cell line N2a (Antonin et al., 2000; Pryor et al., 2004; Itakura et al., 2012; Pilliod et al., 2020). Other groups have also demonstrated that the endosomal system contributes to Tau secretion. In a recent study, it was reported that Bin1(bridging integrator 1), a protein involved in endocytosis and subcellular trafficking can bind to Tau and regulate its secretion (Prokic et al., 2014; Glennon et al., 2020). Its loss resulted in a significant decrease of Tau secretion by neurons. Interestingly, polymorphisms associated with Bin1 is the second largest genetic risk for sporadic AD (Lambert et al., 2013; Vardarajan et al., 2015). Syntaxins 6 and 8, two SNAREs that play an important role in the membranes trafficking, can interact with Tau C-terminal and increase its secretion (Lee et al., 2021). Syntaxin 6 is found at the trans-Golgi network and early endosomes whereas syntaxin 8 is localized on recycling and late endosomes (Jung et al., 2012).

The above observations revealed that Tau can be secreted by several pathways. In both the CSF and culture medium of neuronal cells, full length-Tau (FL-Tau) and N- and C-terminal truncated forms are detected (Mohamed et al., 2013; Pernegre et al., 2019). It remains unclear whether these forms of Tau are released by distinct secretory pathways. The amount of FL-Tau released by primary neuronal cultures varies from one study to another. In some studies, it was the main form whereas in other studies it was a minor pool of secreted Tau (less than 1%) (Plouffe et al., 2012; Pooler et al., 2013; Mohamed et al., 2014; Bright et al., 2015; Kanmert et al., 2015; Mohamed et al., 2017; Rodriguez et al., 2017).

The above observations revealed that different cleaved forms of Tau are released by neurons. We previously reported that VAMP8 increases Tau secretion, and that secreted Tau was cleaved at the C-terminal (Pilliod et al., 2020). In the present study, we examined whether the increase of Tau secretion by VAMP8 affected its intra- and extracellular cleavage. Upon VAMP8 overexpression, an increase of Tau cleaved by caspase-3 in the cell lysate and in the medium was observed which, was correlated to an increase of active caspase-3 in the cell lysate and the medium. However, our results revealed that Tau cleavage by caspase-3 was not necessary for its secretion upon VAMP8 overexpression. We also demonstrated that the asparagine N410 affected the cleavage of secreted Tau at the C-terminal when VAMP8 was overexpressed. Lastly, we demonstrated that N-terminal of Tau regulated the secretion pattern of a Tau fragment containing the microtubule-binding domain and the C-terminal of Tau upon VAMP8 overexpression. All above observations indicate that VAMP8 influences the intra- and extracellular cleavage pattern of Tau.

## Materials and methods

### Cell culture

Neuro-2A cells were purchased from ATCC (#CCL-131TM, Manassas, VA, United States) and were cultured in MEM with Earles’s Salt, non-essential amino acids supplemented with L-glutamine, Na-pyruvate and Na-bicarbonate (#320-026-CL, Wisent Life Sciences, Saint-Bruno, QC, CANADA) and with 10% foetal bovine serum premium (Wisent, Saint Bruno, QC, CANADA) at 37°C in a humidified 5% CO2 incubator.

### Chemicals, antibodies, and plasmids

The protease inhibitor cocktail, cOmplete™ ULTRA tablets from Roche Diagnostics was used (#5892988001, Roche Diagnostics, Indianapolis, IN, United States). For immunoblotting, the following antibodies were used : total Tau (1:50000 #A0024, Dako, Santa Clara, CA, United States); GFP (1:1000 #3H9, Chromotek Inc., Hauppauge, NY, United States); ɣ-actin (1:10000 #Sc-65635, Santa Cruz, Dallas, TX, United States); Tau-46 (1:500 #ab203179, Abcam, Cambridge, MA, United States); Tau-C3 (1:1000 #AHB0061, Invitrogen, Carlsbad, CA, United States); Caspase3 (1:1000 #9662, CellSignaling); Cleaved Caspase3 (1:1000 #9661, CellSignaling); α-Synuclein (1:1000 #610787, BD Biosciences, Franklin Lakes, NJ, United States). All the secondary antibodies were coupled with HRP from Jackson ImmunoResearch (West Grove, PA, United States). The Flag-Tau, Flag-empty, GFP-VAMP8 and GFP-empty plasmids used for co-transfection of Neuro-2A cells were described previously (Pilliod et al., 2020). Tau-D421A, Tau-Δ421-441, Flag-TauNT and Flag-TauMBD-CT were generated by mutagenesis from Flag-Tau and Tau-N410A+D421A from pEGFP-C1-4R-Tau by removing the GFP tag (Civic Biosciences limitée, Beloeil, QC, Canada). Myc- α-synuclein was obtained from Dr. EA Fon. Recombinant Tau protein (0N4R) was obtained from Bio-techne (SP-499, Bio-Techne, Minneapolis, MN, United States).

### Plasmid transfection

Neuro-2A cells were plated into 35 mm plates and transfected the next day with plasmids using Genejuice (#70967, Millipore-Sigma, ON, Canada) and 48 h post-transfection cells were lysed for immunoblotting.

### Extracellular cleavage of tau assay

To determine whether Tau can be cleaved extracellularly by caspase-3, the medium either of untransfected or transfected cells was collected 48 h post transfection and transferred to a petri dish without cells. 1.5 µg of recombinant Tau with or without protease inhibitors was added to the medium and the dishes were placed in the incubator for 12 h. The protease inhibitors were prepared according to the manufacturer instructions.

### Western blot

The culture medium of N2a cells was collected 48 h after transfection and centrifuged at 3,000 rpm for 10min at 23°C to remove cell debris. For cell lysates, cells were washed twice with PBS and once with PBS containing 0.5 M NaCl and lysed in fresh lysis buffer containing Tris 50mM, NaCl 300mM, Triton 100 × 0.5%, a protease inhibitor cocktail (cOmplete™ ULTRA tablets), and a phosphatase inhibitor cocktail (PhosSTOP, Roche Diagnostics), and then incubated on ice for 20 min. Proteins were quantified using Bio-Rad DC Protein assay (Bio-Rad Laboratories Ltd., Mississauga, ON, Canada). The medium and the lysates were mixed with Laemmli buffer 1X and boiled for 5 min at 95°C. Equal amount of the culture medium and cell lysates were loaded in each lane and electrophoresed on polyacrylamide gel. Immunoblotting was performed as previously described (Plouffe et al., 2012). All the secondary antibodies purchased from Jackson ImmunoResearch were coupled with HRP. The quantification of the immunoreactive bands from western blot image acquisition was performed using a ChemiDoc MP system (Bio-Rad Laboratories) and densitometry analysis was done with Image Lab software (version 5.0, Bio-Rad Laboratories).

### Calculation of normalized tau secretion

In all the graphs presenting the quantification of Tau secretion by western blotting, normalized Tau secretion was calculated by dividing the signal of total Tau in the medium (ExTau) by the signal of total Tau in the cell lysate (InTau). The signal of InTau was normalized to that of actin in the cell lysate.

### LDH assay

Lactase dehydrogenase activity (LDH) in media was determined using a LDH Activity Assay Kit (Cayman Chemical Company, Ann Arbor, MI, United States) according to manufacturer instructions. The LDH was measured using a BIO-TEK SYNERGY4 plate reader at Abs 490 nm (Winooski, VT, United States). The mean of the enzyme activity was used for comparison between experimental conditions.

### Statistical analysis

**FIGURE 1:**
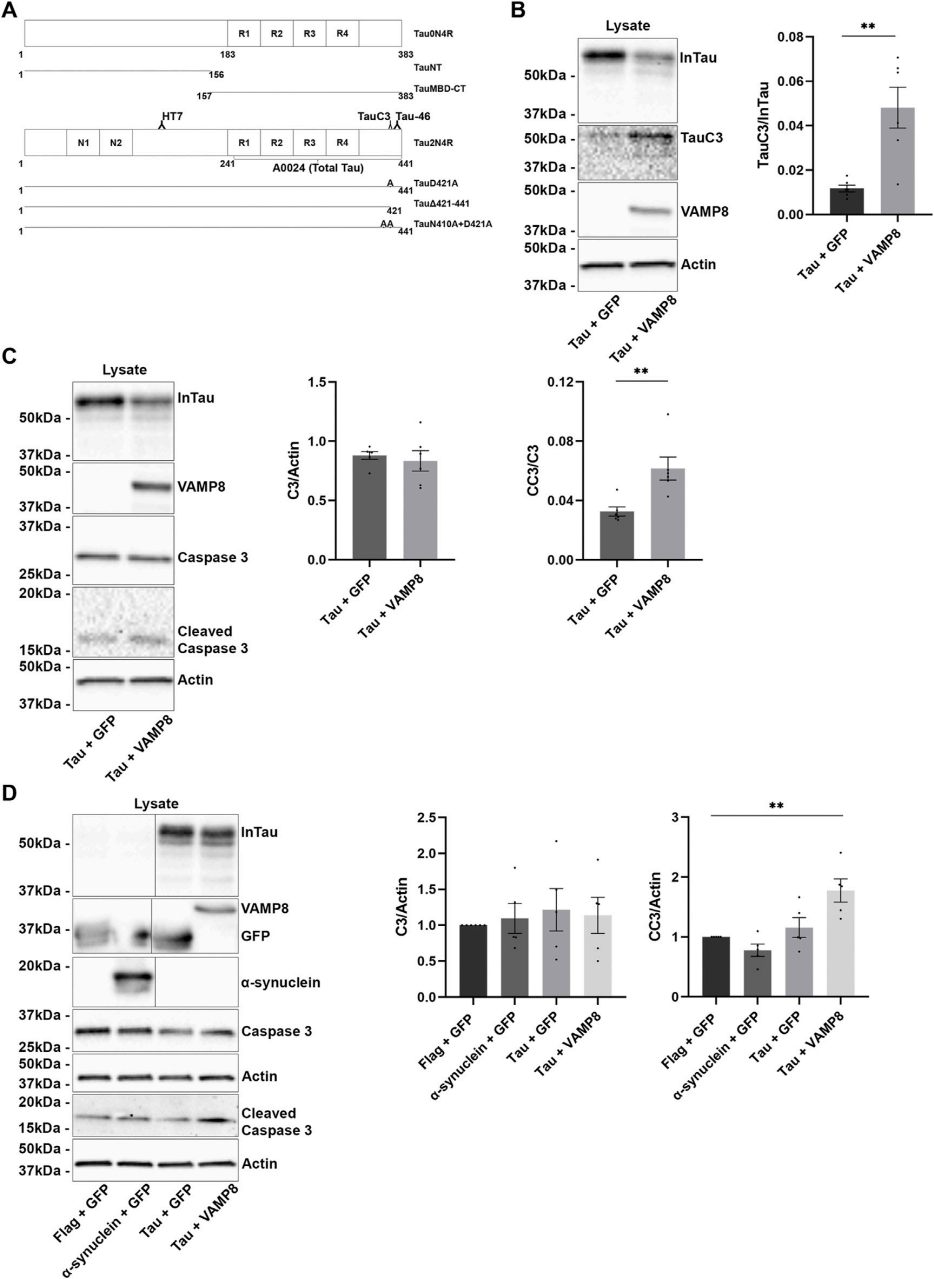
Overexpression of VAMP8 increases the cleavage of Tau by Caspase 3 and increases the amount of active caspase-3 in the cell lysate. For all the figures, GFP alone corresponds to GFP-empty vector. **(A)** Schematic representation of Tau constructs and Tau antibodies. All the experiments were carried out with 0N4R Tau isoform. The constructs TauNT and TauMBD-CT were produced from 0N4R. For the antibodies, the isoform 2N4R was used for consistency with the literature. For B and C, N2a cells were transfected either with Flag-Tau and GFP-empty or Flag-Tau and GFP-VAMP8 plasmids. **(B)** Representative Western blot with the anti-Tau antibody A0024 recognizing total intracellular Tau (InTau) and the antibody TauC3 directed against Tau cleaved by caspase-3 of the cell lysate revealing that the overexpression of VAMP8 decreased InTau but increased InTau cleaved by caspase-3 (TauC3). For the densitometry analysis of the TauC3/total Tau ratio, InTau (total Tau) was normalized with the actin signal, the loading reference. **(C)** Representative Western blot with the anti-Tau antibody A0024, the anti-caspase-3 antibody (C3) and the anti-cleaved caspase-3 antibody (CC3) of the cell lysate showing that the C3 signal is similar for cells overexpressing either Tau alone or Tau and VAMP8 while the co-expression of Tau and VAMP8 increased intracellular CC3. CC3/C3 ratio was analyzed by densitometry. *n* = 6. Data represent scatter plot and mean±SEM. **p < 0.01. **(D)** N2a cells were transfected either with Flag-empty and GFP-empty (Flag + GFP), α-synuclein and GFP-empty, Flag-Tau and GFP-empty, Flag-Tau and GFP-VAMP8 plasmids. Representative Western blot of the cell lysate revealed with the antibodies recognizing Tau (A0024), VAMP8, GFP, α-synuclein, caspase-3, cleaved caspase-3 and actin. No difference of total caspase-3 levels was noted between the experimental conditions. In the case of cleaved by caspase-3, the condition Tau and VAMP8 presented the highest levels. For the densitometry analysis of the signal of caspase-3 and cleaved caspase-3 was normalized with the actin signal, the loading reference. Black frames were used to mark the splice sites of immunoblot images. *n* = 5. Data represent scatter plot and mean±SEM. **p < 0.01.

**FIGURE 2:**
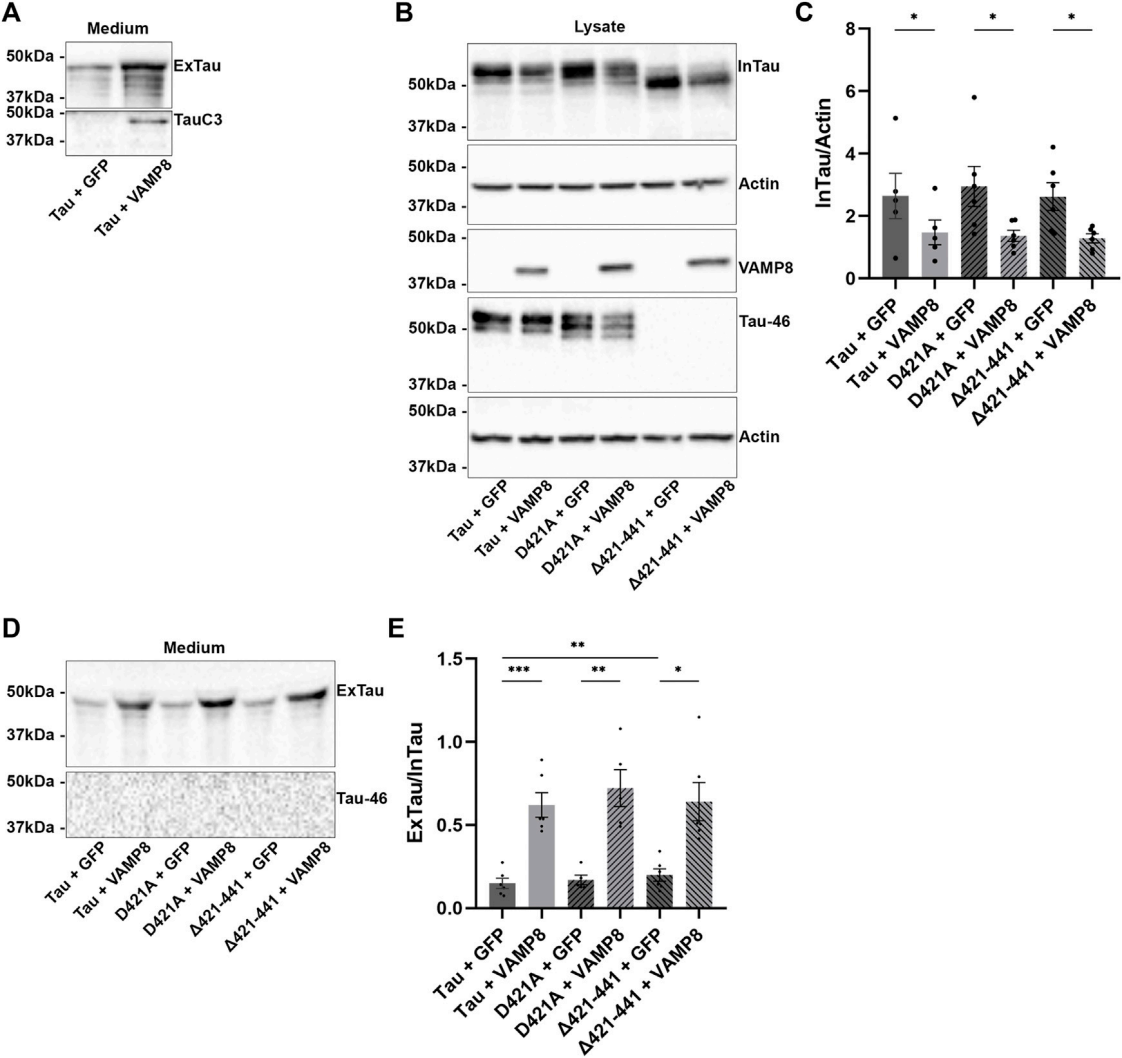
Secretion of Tau upon VAMP8 overexpression does not depend on the cleavage of Tau by caspase-3. N2a cells were transfected either with Flag-Tau and GFP-empty, Flag-Tau and GFP-VAMP8, TauD421A and GFP-empty, TauD421A and GFP-VAMP8, TauΔ421-441 and GFP-empty or TauΔ421-441 and GFP-VAMP8 plasmids for 48 h. **(A)** Representative Western blot with TauC3 antibody of the medium showing the detection of Tau cleaved by caspase-3 only detectable with VAMP8 overexpression. **(B)** Representative Western blot with A0024 of the cell lysate showing that the overexpression of VAMP8 decreased intracellular Tau (InTau) of all Tau mutants. **(C)** Densitometry analysis of A0024 signal of InTau. *n* = 6. Data represent scatter plot and mean±SEM. *p < 0.05. **(D)** Representative Western blot with A0024 of the medium showing that the overexpression of VAMP8 increased extracellular Tau (ExTau) of all Tau mutants. **(E)** Densitometry analysis of A0024 signal of the ExTau/InTau ratio. *n* = 6. Data represent scatter plot and mean±SEM. *p < 0.05, **p < 0.01, ***p < 0.001.

**FIGURE 3:**
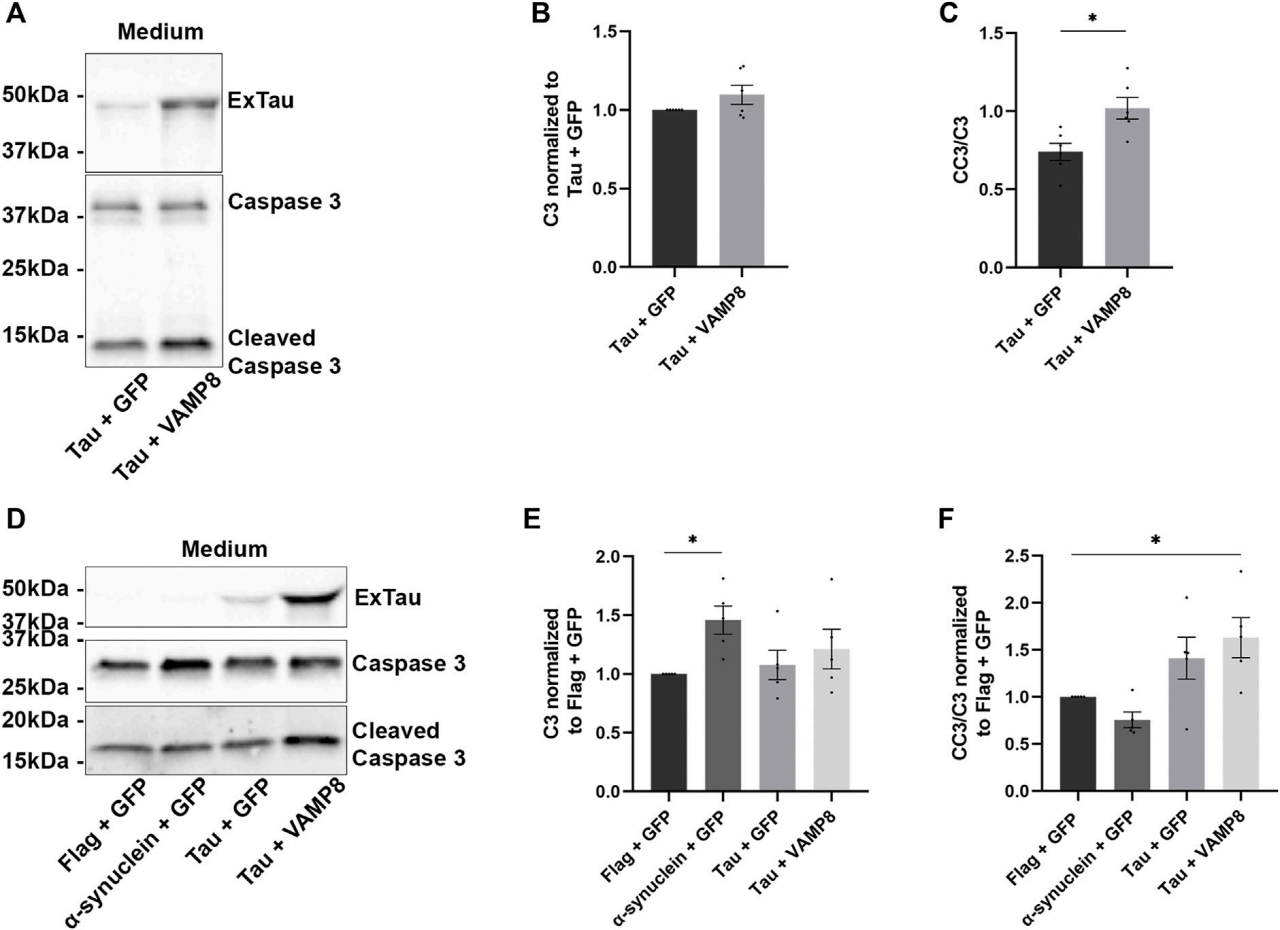
Increased secretion of active caspase-3 upon the overexpression of Tau and VAMP8. **(A)** N2a cells were transfected either with Flag-Tau and GFP-empty or Flag-Tau and GFP-VAMP8 plasmids for 48 h. Representative Western blot with A0024 for staining of extracellular Tau (ExTau), the antibody C3 for total caspase-3 and the antibody CC3 for cleaved caspase-3 of the medium revealing that the secretion of total caspase 3 was similar with Tau alone or Tau and VAMP8 overexpression and that cleaved caspase-3 was increased for Tau and VAMP8. **(B,C)** Densitometry analysis of C3 and CC3/C3 ratio in the medium. The signal of C3 and CC3 was normalized with that of Tau and GFP. *n* = 6. Data represent scatter plot and mean ± SEM. *p < 0.05. **(D)** N2a cells were co-transfected either with Flag-empty and GFP-empty, α-synuclein and GFP-empty, Flag-Tau and GFP-empty or Flag-Tau and GFP-VAMP8 plasmids. Representative Western blot of the medium to confirm the secretion of Tau, caspase-3 and cleaved caspase-3. **(E,F)** Densitometry analysis of C3 and CC3 signals normalized to that of Flag-empty + GFP-empty. The secretion of CC3 in the medium of Flag-tau and GFP-VAMP8 was significantly higher than that detected in Flag-empty + GFP-empty. N = 5. Data represent scatter plot and mean±SEM. *p < 0.05.

**FIGURE 4:**
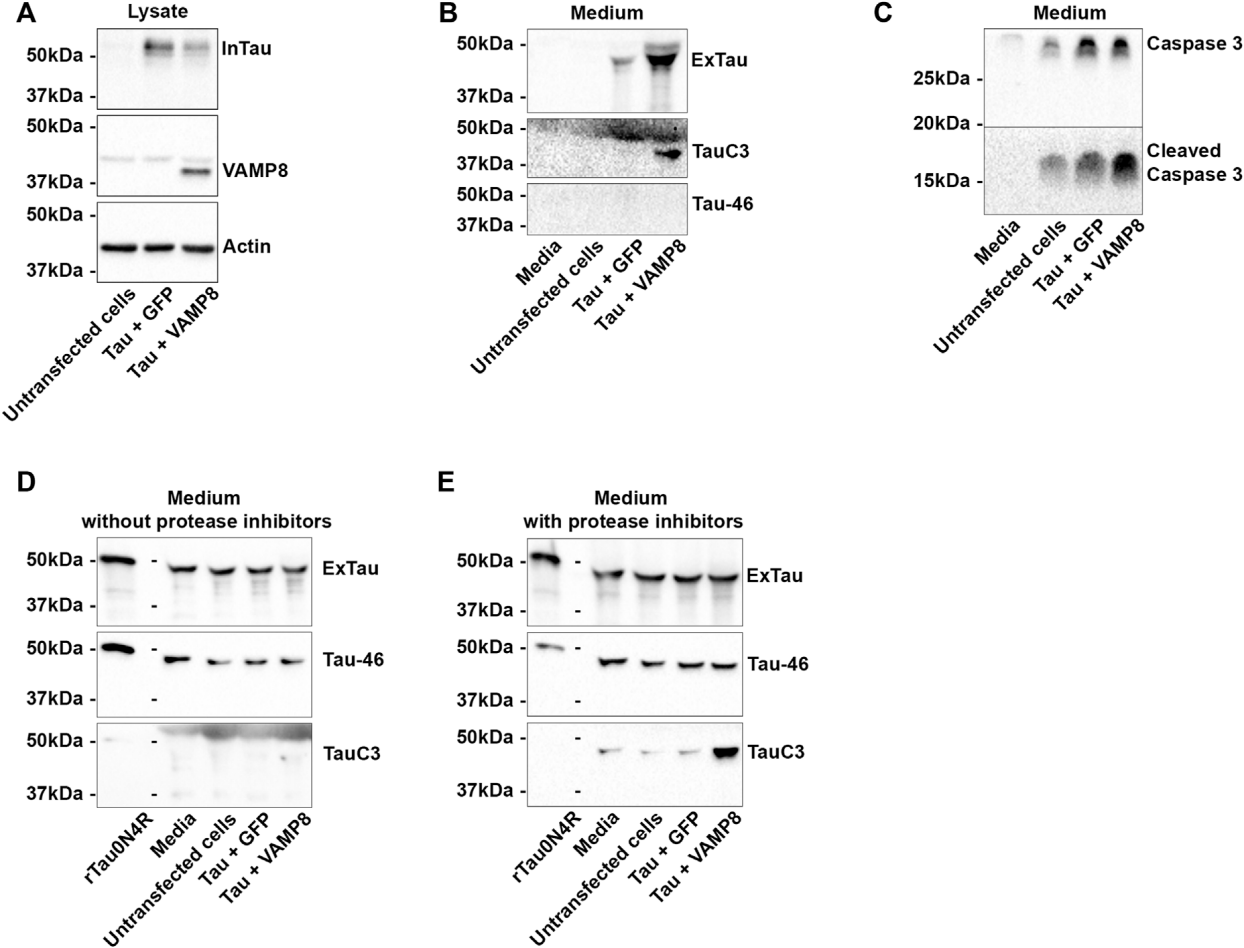
Extracellular cleavage of Tau by caspase-3. N2a cells were transfected either with Flag-Tau and GFP-empty or Flag-Tau and GFP-VAMP8 plasmids. Recombinant 0N4R Tau protein (rTau) was added in media after 48 h post transfection. The medium without cells and the medium of untransfected cells were used as controls. **(A)** Representative Western blot of the cell lysate confirming the expression of Tau and VAMP8. **(B)** Representative Western blot of the medium showing the secretion of Tau and Tau cleaved by caspase-3 (TauC3). The lack of signal with the anti-Tau antibody Tau-46 indicated that Tau was cleaved at the C-terminal. **(C)** Representative Western blot with anti-caspase-3 and the anti-cleaved caspase-3 antibodies to confirm their presence in the medium before the addition of rTau. **(D)** Representative Western blot with the anti-Tau antibodies A0024, TauC3 and Tau-46 after addition of rTau showing that the presence of Tau full-length as revealed by Tau46 antibody. A weak band was detected with TauC3 indicating cleavage of rTau by caspase-3. **(E)** Representative Western blot with the anti-Tau antibodies A0024, TauC3 and Tau-46 after addition of rTau and protease inhibitors showing the important increase of TauC3 signal. N = 3.

**FIGURE 5:**
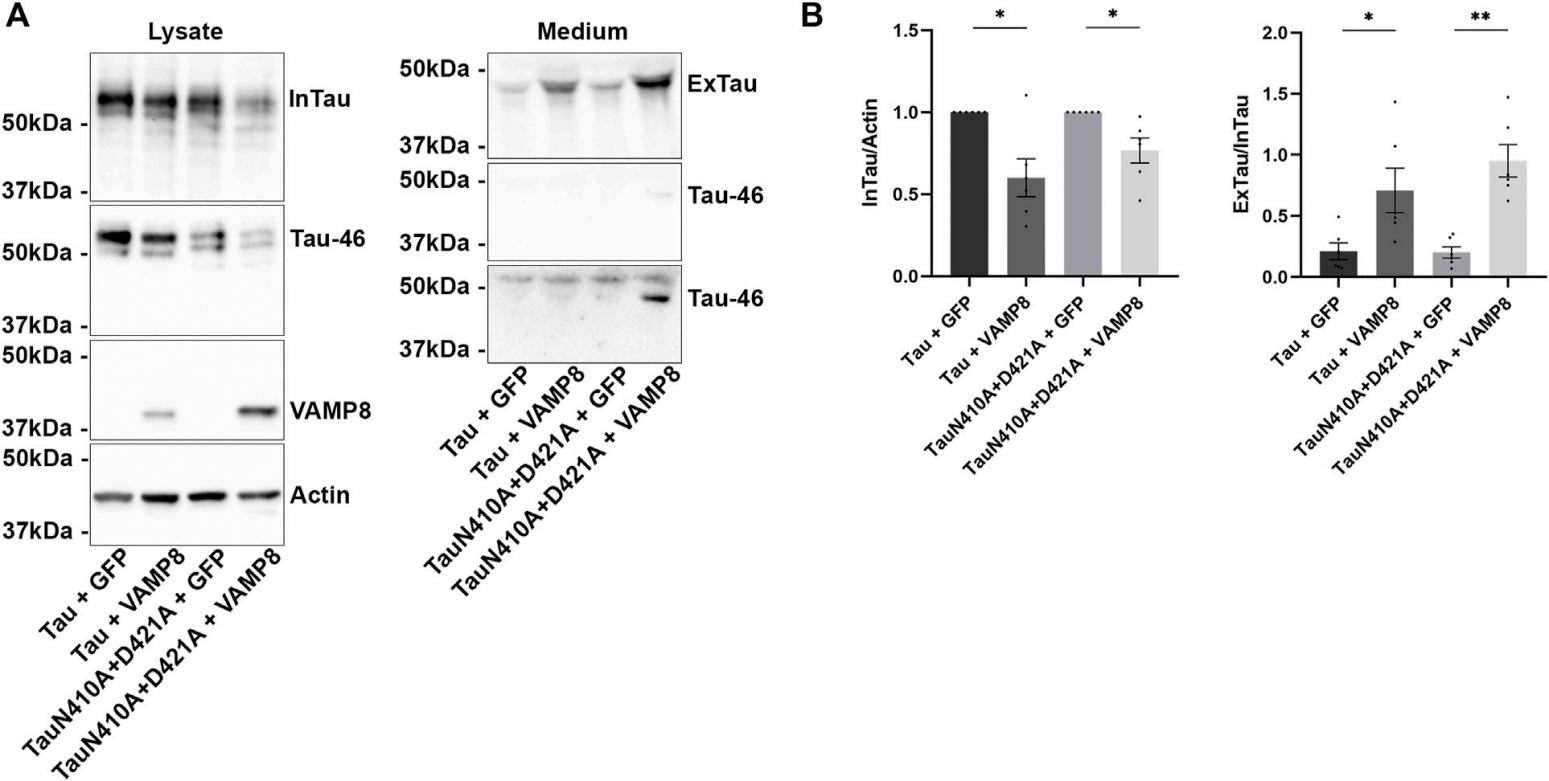
The asparagine N410 affects the cleavage of secreted Tau upon VAMP8 overexpression. N2a cells were transfected either with Flag-Tau and GFP-empty, Flag-Tau and GFP-VAMP8, TauN410A+D421A and GFP-empty or TauN410A+D421A and GFP-VAMP8 plasmids for 48 h. **(A)** Representative Western blot with A0024 of the cell lysate and the medium revealing that the overexpression of VAMP8 decreased intracellular Tau (InTau) and increased extracellular Tau (ExTau) for both Tau mutant and wild-type. A signal was detected with the anti-Tau antibody Tau46 in both the cell lysate and medium indicating the presence of uncleaved Tau at the C-terminal. A short and a long exposure times are presented to show that the Tau46 antibody signal was not detected when wild-type Tau was expressed. **(B)** Densitometry analysis of A0024 signal of InTau. Actin was used as a loading reference. Densitometry analysis of A0024 signal of ExTau. Normalized Tau secretion corresponds to the ratio ExTau/InTau. InTau was normalized with the actin signal. N = 6. Data represent scatter plot and mean±SEM. *p < 0.05. **p < 0.01.

**FIGURE 6:**
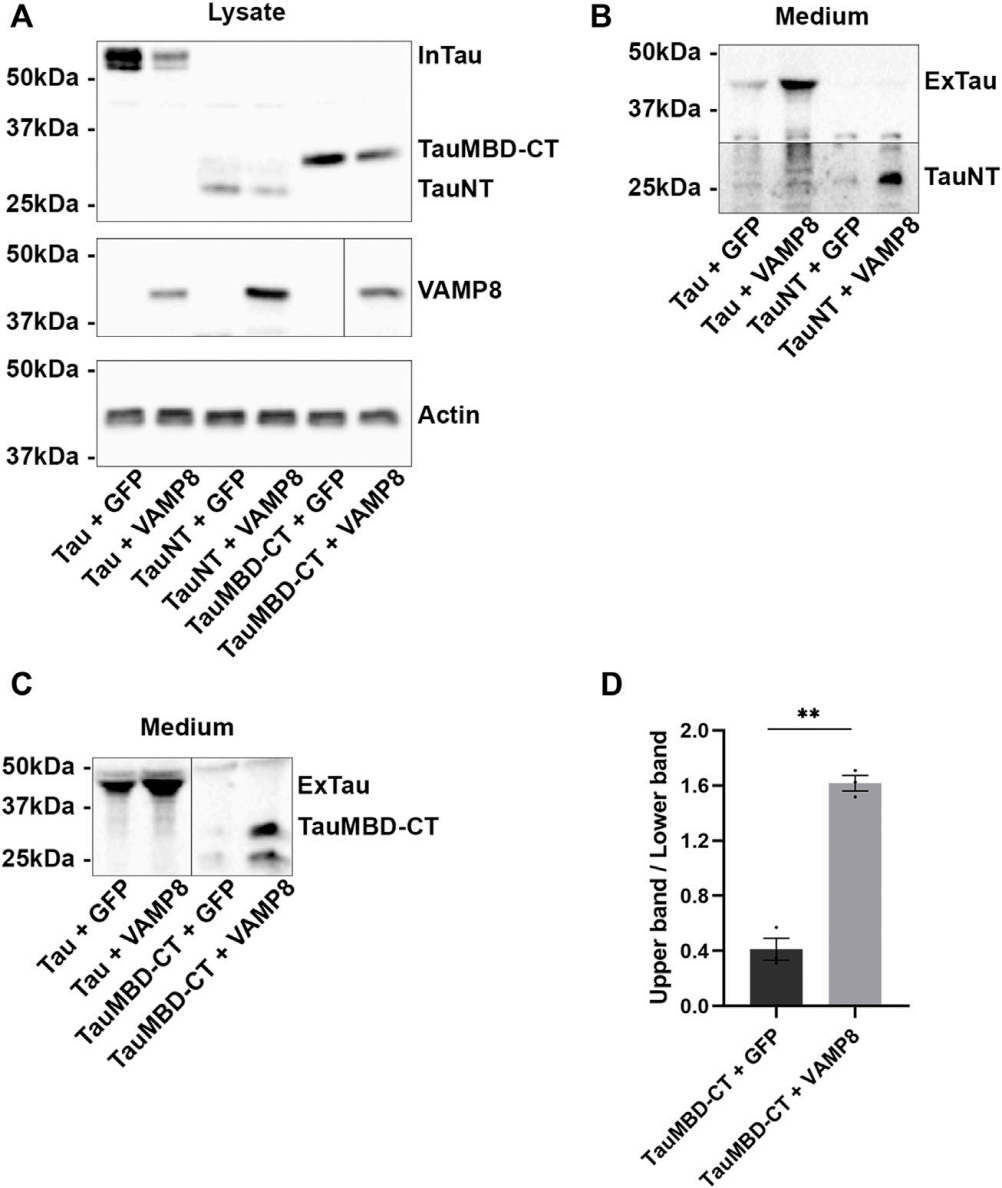
Secretion of Tau mediated by VAMP8 is independent of C-terminal cleavage. N2a cells were transfected either with Flag-Tau and GFP-empty, Flag-Tau and GFP-VAMP8, Flag-TauNT and GFP-empty, Flag-TauNT and GFP-VAMP8, Flag-TauMBD-CT and GFP-empty or Flag-TauMBD-CT and GFP-VAMP8 plasmids for 48 h. **(A)** Representative Western blot with the anti-FLAG antibody for intracellular signal of Tau, TauNT and TauMBD-CT. **(B)** Representative Western blot with the anti-Tau antibody HT7 to reveal extracellular TauNT. **(C)** Representative Western blot with the anti-Tau antibody A0024 to reveal extracellular TauMBD-CT. **(D)** Densitometry analysis of the upper band/lower band ratio for TauMBD-CT. Black frames were used to mark the splice sites of immunoblot images. *n* = 3. Data represent scatter plot and mean±SEM. **p < 0.01.

The statistical analysis was performed using Prism 8.0c software (GraphPad Software Inc., San Diego, CA, United States). Normality was assumed for the statistical analysis. Findings were considered significant as follows: *p < 0.05, **p < 0.01, ***p < 0.001, or ****p < 0.0001. When we compared the means of 3 or more experimental groups (Figures 1, 3), statistical significance was evaluated with an ordinary one-way ANOVA test. The experimental groups were compared to the control group. When the means of two groups were compared, a paired *t*-test was used (Figures 2, 4–6).

## Results

### Intracellular and extracellular tau is cleaved by caspase-3 upon VAMP8 overexpression

We previously showed that VAMP8 overexpression increased Tau secretion in the neuroblastoma cell line N2a, which was correlated to a decrease of intracellular Tau (Pilliod et al., 2020). Secreted Tau induced by VAMP8 overexpression was cleaved at the C-terminal as revealed by the lack of staining with the anti-Tau antibody, Tau46 that does not recognize Tau cleaved between the 404–441 amino acids located at its C-terminal (Figure 1A). Full-length intracellular Tau was recognized by the antibody Tau46 but no signal was detected in the medium revealing that secreted Tau was cleaved at the C-terminal (Pilliod et al., 2020). In the present study, we examined the intracellular and extracellular forms of cleaved Tau upon VAMP8 overexpression. N2a cells were co-transfected either with Flag-Tau and GFP-empty or Flag-Tau and GFP-VAMP8 plasmids as previously described (Pilliod et al., 2020). Interestingly, an increase of Tau cleaved by caspase-3 was observed in the cell lysate of cells overexpressing VAMP8 indicating that caspase-3 was activated in these cells (Figure 1B). To demonstrate that it was the case, we examined the protein levels of total caspase-3 and cleaved caspase-3, its active form, in the cell lysate of cells overexpressing either Tau alone or Tau and VAMP8. The amount of total caspase-3 was similar in the cell lysate of cells overexpressing either Tau alone or Tau and VAMP8, but a significant increase of cleaved caspase-3 was observed in cells overexpressing Tau and VAMP8 compared to cells overexpressing Tau alone (Figure 1C). This increase of active caspase-3 in the lysate of cells overexpressing Tau and VAMP8 was consistent with the increase of Tau cleaved by caspase-3 in these cells. We then examined whether the increase of active caspase-3 was specific to Tau and VAMP8 overexpression. To do so, cells were co-transfected either with GFP-empty and Flag-empty (the empty plasmids of VAMP8 and Tau, respectively), GFP-empty and α-synuclein (a secreted protein linked to Parkinson’s disease) or Tau and VAMP8 (Figure 1D). No difference between these different conditions was noted in the amount of total caspase-3. In the case of active caspase-3, only the condition Tau and VAMP8 was statistically different from the control condition, GFP-empty and Flag-empty.

We then examined whether secreted Tau was also cleaved by caspase-3 upon VAMP8 overexpression. As noted for intracellular Tau, extracellular Tau was cleaved by caspase-3 (Figure 2A). No Tau cleaved by caspase-3 was detected in the medium of cells overexpressing Tau alone. The LDH was measured to monitor cell death. No difference was noted between the different conditions indicating that extracellular Tau was not released by cell death (Supplementary Figure S1).

### Tau cleavage by caspase-3 is not necessary for its secretion upon VAMP8 overexpression

The above results prompted us to examine whether cleavage of Tau by caspase-3 was necessary for Tau secretion upon VAMP8 overexpression. To test this, a Tau mutant either mimicking Tau cleavage by caspase-3 (Δ421-441) or not cleavable by caspase-3 (D421A) was produced and co-expressed with VAMP8 in N2a cells. The expression of these Tau mutants was confirmed by WB (Figure 2B). The antibody Tau46 did not reveal the mutant Δ421-441 as expected (Figure 2B). A decrease of intracellular D421A and Δ421-441was noted when VAMP8 was overexpressed as observed for wild-type Tau (Figures 2B,C). Consistent with this, an increase of extracellular D421A and Δ421-441 similar to that of wild-type Tau was found upon VAMP8 overexpression (Figure 2D). As noted in our previous study, Tau mutant mimicking its cleavage by caspase-3 was more secreted than wild-type Tau although this effect was less important than that previously observed in Hela cells (Figures 2D,E) (Plouffe et al., 2012). Interestingly, the mutant non-cleavable by caspase-3, D421A, was cleaved at the C-terminal by another protease as revealed by the lack of staining with the Tau46 antibody (Figure 2D). These results demonstrated that cleavage by caspase-3 was not necessary for Tau secretion by VAMP8.

### Extracellular cleavage of tau by caspase-3 upon VAMP8 overexpression

The fact that cleavage of Tau by caspase-3 was not necessary for its secretion by VAMP8 prompted us to examine the possibility that Tau could be extracellularly cleaved by caspase-3. Indeed, caspase-3 was previously shown to be secreted by cells (Garcia-Faroldi et al., 2013; Zorn et al., 2013). We examined the amount of total caspase-3 and cleaved caspase-3 in the medium. The amount of total caspase-3 in the medium was similar for cells either overexpressing Tau alone or Tau and VAMP8 (Figures 3A,B). In the case of active caspase-3, the cells overexpressing Tau and VAMP8 presented higher levels than the cells overexpressing Tau alone (Figure 3C). We then verified whether the increase of active caspase-3 in the medium was specific to Tau and VAMP8. To do so, we compared the amount of total caspase-3 and active caspase-3 in the medium of cells co-overexpressing the control plasmids (GFP-empty and Flag-empty), GFP-empty and α-synuclein, Flag-Tau and GFP-empty and Flag-Tau and GFP-VAMP8. The amount of total caspase-3 was similar to that of the control plasmids for all the experimental conditions except for the condition α-synuclein and GFP-empty presenting a higher amount than control plasmids (Figures 3D,E). In the case of active caspase-3, only the condition Tau and VAMP8 was statistically different from the control plasmids (Figures 3D,F). The fact that caspase-3 and its active form could be found in the medium of N2a cells indicated that Tau cleavage could occur in the medium.

To further demonstrate that Tau cleavage by caspase-3 could occur in the medium, recombinant human Tau (0N4R) (rTau) was added either to the medium without cells, medium of untransfected cells and medium of cells transfected either with Tau alone or Tau and VAMP8. Two sets of experiments were carried out. In the first set, the medium was collected after 48 h of transfection and transferred to a petri dish without cells. Transfection of Tau and VAMP8 and Tau secretion were confirmed by WB (Figures 4A,B). The presence of caspase-3 and cleaved caspase-3 in the medium was also confirmed by WB (Figure 4C). For all the experiments, rTau (1.5 μg) was added for 12 h in the medium. Most of the signal detected by the anti-Tau antibody A0024 in the medium corresponded to rTau. Indeed, the signal was stronger when rTau was added compared to that obtained for medium only containing secreted Tau when the membranes were revealed side by side (Supplementary Figure S2). Furthermore, the fact that the intensity of Tau signal was similar in medium containing or not secreted Tau also indicated that most of the anti-Tau antibody signal corresponded to rTau. Lastly, the signal of the anti-Tau antibody Tau46 in the medium, which did not reveal secreted Tau, confirmed that the main signal was generated by rTau. FL-Tau revealed by the anti-Tau antibodies, Tau46 and A0024, and fragments of rTau mainly detected with the antibody A0024 were observed (Figure 4D). This indicated that some degradation and/or cleavage had occurred in the medium. A weak staining with the anti-Tau antibody TauC3 recognizing Tau cleaved by caspase-3 was noted in the medium collected from cells overexpressing Tau and VAMP8, which contained the highest levels of active caspase-3 as shown in Figure 3C (Figure 4D). The number of Tau bands was higher in the medium obtained either from untransfected cells, cells overexpressing Tau or cells overexpressing Tau and VAMP8 than in the medium not incubated with cells. This indicated that proteases were released by cells with and without transfection. In the second set of experiments, rTau and protease inhibitors were added to the medium. The protease inhibitors were directed against metalloproteases and serine and cystein proteases (Roche Diagnostics). Interestingly, a lower number of Tau-positive bands was detected in the medium indicating that Tau was less cleaved by these proteases (Figure 4E). Furthermore, this was correlated to an increase of TauC3 staining in the medium collected from the cells overexpressing Tau and VAMP8. This indicated that Tau was cleaved by caspase-3 in the medium as well as by metalloproteases and/or cysteine and serine proteases.

### Asparagine 410 affects the cleavage of secreted tau upon VAMP8 overexpression

Tau was still cleaved in the medium when the site of caspase-3 was mutated to prevent its cleavage as revealed by the lack of staining with the antibody Tau46 for this mutant (Figure 2D). Our previous study revealed that secreted Tau could be cleaved at a site in close vicinity to the phosphorylation site S409 (Plouffe et al., 2012). Tau is known to be cleaved by asparagine endopeptidase (AEP) at the C-terminal (Zhang et al., 2014). Based on this, the asparagine 410 (N410), which could be a potential cleavage site by AEP, was mutated to alanine to prevent its cleavage in the mutant non-cleavable by caspase-3 (N410A+D421A). Interestingly, a recent study reported that N410 can be glycosylated and can modulate Tau pathology (Losev et al., 2021). The secretion of N410A+D421A mutant was tested in N2a cells upon VAMP8 expression. Its secretion was similar to that of wild-type Tau indicating that the cleavage and glycosylation of N410 was not necessary for Tau secretion (Figures 5A,B). In contrast to Tau mutant only resistant to cleavage by caspase-3 (D421A), a weak band reactive to the antibody Tau46 was detected in the medium of N410A+D421A mutant indicating that a portion of secreted Tau was not cleaved at the C-terminal upon VAMP8 overexpression. The above results revealed that the N410 could alter the cleavage of Tau found in the medium upon VAMP8 overexpression.

### N-terminal deletion of tau modifies its pattern of secretion upon VAMP8 overexpression

The above results revealed that secreted Tau could be cleaved at the C-terminal by different proteases, but these cleavage events did not have significant impact on Tau secretion upon VAMP8 overexpression. We then asked whether the N-terminal deletion could exert regulatory effects on Tau secretion by VAMP8. To investigate this point, we produced two Tau mutants, one containing Tau N-terminal (TauNT) and one containing Tau microtubule-binding domain and its C-terminal (TauMBD-CT) (Figure 1A). The secretion of TauNT was increased upon VAMP8 overexpression as noted for FL-Tau (Figures 6A,B). Its pattern was similar to that of FL-Tau meaning one main band was detected in the culture medium. Interestingly, the secretion pattern of TauMBD-CT was different upon VAMP8 overexpression compared to that of its overexpression alone (Figure 6C). When it was overexpressed in the absence of VAMP8, two bands were detected in the medium, a very weak upper band and a stronger lower band as revealed with the anti-Tau antibody A0024. Upon the overexpression of VAMP8, this pattern was inverted (Figures 6C,D). Collectively, the above observations indicate that the N-terminal has an effect of the pattern of Tau secretion upon VAMP8 overexpression since its deletion resulted in a different pattern of TauMBD-CT secretion.

## Discussion

In the present study, we examined whether the increase of Tau secretion by VAMP8 affected its intra- and extracellular cleavage. Upon VAMP8 overexpression, an increase of Tau cleaved by caspase-3 in the cell lysate was observed. This increase was correlated to an increase of active caspase-3. Using a Tau mutant not cleavable by caspase-3, we demonstrated that Tau cleavage by caspase-3 was not necessary for its secretion upon VAMP8 overexpression. We also demonstrated that Tau cleavage by caspase-3 could occur extracellularly because of the secretion of active caspase-3 by cells overexpressing Tau and VAMP8. Our results also revealed that N410 affected the cleavage of Tau released upon VAMP8 overexpression. Lastly, we observed that the N-terminal of Tau regulated the secretion of a Tau fragment containing the microtubule-binding domain and C-terminal upon VAMP8 overexpression.

Our results demonstrated that the cells overexpressing VAMP8 presented the highest levels of active caspase-3 in the cell lysate. A previous study reported that the protein levels of VAMP8 was regulated by caspases in dendritic cells (Ho et al., 2009). The inhibition of caspases increased its protein levels. Based on this, the increase of active caspase-3 observed in our experimental conditions could be a protective reaction to prevent an excessive overexpression of VAMP8. This increase of active caspase-3 was correlated to an enhanced cleavage of Tau in the cell lysate. In most studies, Tau cleaved by caspase-3 was found to be detrimental to neurons and to contribute to Tau pathology (Means et al., 2016; Cieri et al., 2018). It was also associated with the progression of AD (Basurto-Islas et al., 2008; Jarero-Basulto et al., 2013; Zhou et al., 2018). However, recent studies indicate that Tau cleavage by caspase-3 could be neuroprotective. A study reported that at the early stages of AD, caspase-3 was activated without leading to neuronal cell death (de Calignon et al., 2010). More recently, a study demonstrated that in mice, blocking Tau cleavage by caspase-3 resulted in memory deficits (Biundo et al., 2017). In *Drosophila*, caspase 3 cleavage of hyperphosphorylated Tau prevented its toxicity and allowed recovery of motor deficits (Chi et al., 2020). From these results, it appears that the increased cleavage of Tau by caspase-3 upon VAMP8 overexpression could be neuroprotective. In such a case, VAMP8 would decrease toxicity of intracellular Tau by this cleavage.

We found that VAMP8 increased the secretion of active caspase-3. It was previously demonstrated that removing active caspase-3 through lysosomal degradation was linked to an increase of survival of neuronal cells induced by NGF (Mnich et al., 2014). VAMP8-induced secretion of active caspase-3 was also shown to be protective in the pancreatic acinar cells. The acute inhibition of VAMP8-mediated secretion resulted in the intracellular accumulation of trypsin causing acinar cell damages during pancreatitis (Messenger et al., 2017). Based on these observations, one can postulate that VAMP8 could be part of an unconventional secretory pathway that is beneficial to cells by eliminating proteins that can become toxic and compromise cell survival. Active caspase-3 released upon VAMP8 overexpression seemed to be functional as indicated by the cleavage of rTau. In a previous study, extracellular caspase-3 released by mouse mast cells through secretory lysosomes was reported to be able to cleave interleukin-33 (Zorn et al., 2013). It was proposed that extracellular active caspase-3 could be involved in the processing of cytokines and thereby contributed to the inflammatory response (Garcia-Faroldi et al., 2013). Surprisingly, active caspase-3 was found in the medium of all the conditions that we tested. Furthermore, as revealed by adding protease inhibitors against metalloproteases and serine and cystein proteases in the medium, the number of Tau fragments was reduced indicating that these proteases were present in the medium. This should be considered when examining protease cleavage of extracellular proteins. The present results on Tau cleavage occurring extracellularly does not fit with a previous study reporting no cleavage of Tau in the culture medium of primary neuronal cultures (Kanmert et al., 2015). The authors did not observe any change in the pattern of secreted Tau forms when protease inhibitors were added to the culture medium. The release of proteases could depend on the type of cells. N2a, neuroblastoma cells, might release more proteases than primary neurons. In AD, endosomes were reported to accumulate in the early stages of the disease. Our results with VAMP8 indicate that in such a condition, the release of proteases could be increased, which could contribute to the presence of Tau fragments in the CSF. Indeed, the presence of caspase activity was reported in CSF of patients suffering from dementia and traumatic brain injury (Harter et al., 2001; Albrecht et al., 2009) (Perez-Barcena et al., 2022).

Upon VAMP8 overexpression, FL-Tau and N- and C-terminal fragments of Tau could be released. This corroborates previous studies that reported the release of C-terminal truncated form of Tau by primary neuronal cultures and neurons produced iPSC (Bright et al., 2015; Kanmert et al., 2015). The released of fragments containing the microtubule-binding domain and the C-terminal is more controversial. The lack of detection of such fragments in previous studies could be explained by the fact that the anti-Tau antibody Tau46 was used either for detection by ELISA or for immunoprecipitation in these studies (Bright et al., 2015; Kanmert et al., 2015). In our experiments, this antibody could not detect secreted Tau because of its cleavage at the C-terminal. The mechanisms underlying the pattern of secreted tau fragments remain poorly characterized. In the case of VAMP8-induced secretion, a positive weak signal with the antibody Tau46 in the medium was only detected when N410 was mutated in alanine indicating that this site affected the cleavage of tau at the C-terminal. N410 could be cleaved by an asparagine endopeptidase (Zhang et al., 2014). However, this cleavage remains to be demonstrated. In a recent study, it was reported that N410 can be glycosylated and that preventing it worsened tau pathology (Losev et al., 2021). Our study confirmed the role of this site in tau processing. Our results also demonstrated that the N-terminal had regulatory effects on VAMP8-induced tau secretion given that the secretion pattern of TauMBD-CT, a Tau fragment containing the microtubule binding domain and the C-terminal was different when VAMP8 was overexpressed. In a previous study, it was reported that the N-terminal was necessary for Tau secretion in lamprey (Kim et al., 2010). In this previous study, it was also noted that exon 2 present in the N-terminal exerted inhibitory effects on Tau secretion. Such effects were not confirmed in cultured cells where Tau isoforms containing exon 2 were secreted (Karch et al., 2012). Collectively, the above observations indicate that both the N- and C-terminal can regulate Tau secretion.

Several unconventional secretory pathways are involved in Tau secretion (Mohamed et al., 2013; Pernegre et al., 2019). Our previous studies demonstrated that late endosomes are involved in Tau secretion. We reported that Rab7A, GTPase associated with late endosomes, and VAMP8, a R-SNARE attached to late endosomes, were involved in Tau secretion (Rodriguez et al., 2017; Pilliod et al., 2020). The contribution of VAMP8 to Tau secretion was observed in N2a and neurons. By TIRF microscopy, we observed a depletion of Tau in the cytoplasm upon the fusion of VAMP8-positive vesicles with the plasma membrane (Pilliod et al., 2020). Other proteins involved in the endocytic pathways such as Bin1 and the two SNAREs, syntaxins 6 and 8 also contribute to Tau secretion (Glennon et al., 2020; Lee et al., 2021). It seems possible that each of these pathways could permit the release of a specific set of Tau forms. VAMP8 would induce the release of Tau forms cleaved at the C-terminal. FL-Tau and N- and C-terminal truncated forms were detected in the CSF and culture medium of non-neuronal and neuronal cells (Plouffe et al., 2012; Mohamed et al., 2013; Pooler et al., 2013; Mohamed et al., 2014; Bright et al., 2015; Kanmert et al., 2015; Mohamed et al., 2017; Rodriguez et al., 2017; Pernegre et al., 2019). Exosomes were shown to contain both FL-Tau and C- and N-terminal truncated Tau (Saman et al., 2012; Simon et al., 2012; Dujardin et al., 2014; Guix et al., 2018). In the case of phosphorylation, most studies reported that extracellular membrane-free Tau was less phosphorylated than intracellular Tau (Mohamed et al., 2013; Pernegre et al., 2019). Some discrepancies exist in the literature concerning the phosphorylation levels of exosomal Tau. Indeed, high and low levels were reported (Saman et al., 2012; Wang et al., 2017). Tau oligomers were found to be secreted by translocation across the plasma membrane as well as by exosomes (Saman et al., 2012; Asai et al., 2015; Wang et al., 2017; Merezhko et al., 2018). No Tau aggregates were found to be released by an active process of secretion in the medium where Tau was membrane-free (Kanmert et al., 2015). All together, the above observations indicate that experimental conditions and/or cell types can influence the Tau forms that are released.

Tau secretion could be a mechanism for clearance of Tau, meaning that it is beneficial to neurons by removing toxic forms of Tau. This is supported by recent studies where Tau secretion was shown to reverse of Tau-induced cellular alterations. When Tau secretion was decreased because of Bin1 loss, it resulted in an accumulation of Tau and synaptic dysfunction (Glennon et al., 2020). We showed that the increase of Tau secretion by VAMP8 could reverse the microtubule stability induced by the overexpression of Tau in N2a cells (Pilliod et al., 2020). The different unconventional pathways involved in Tau secretion permit the release of diverse Tau species. It remains to be determined which of these pathways allows the release of toxic forms to prevent their accumulation in neurons and which forms of Tau are toxic in the extracellular space. This information is determinant to elaborate a therapeutic strategy to prevent both the intracellular and extracellular accumulation of toxic Tau species. Tau secretion could be used to increase the accessibility of intracellular Tau species involved in the neurodegenerative process that takes place in AD and FTLD. These species could then be neutralized by a therapeutic agent. Indeed, several undergoing clinical trials target extracellular Tau using an anti-Tau antibody to sequester its toxic species (Jadhav et al., 2019). A therapy combining the increase of Tau secretion with the capture of extracellular toxic Tau species by an antibody could be an efficient approach to prevent the intracellular accumulation of pathological Tau and its propagation in the brain.

## Data Availability

The original contributions presented in the study are included in the article/Supplementary Materials, further inquiries can be directed to the corresponding author.

## Supplementary material

Supplementary Figure S1 LDH data for the experiments on Tau secretion. LDH was measured in the medium for monitoring cell death. The LDH differences (%) between the control condition and the experimental conditions was calculated.

Supplementary Figure S2 Comparaison of the signal of secreted Tau and recombinant Tau in the medium. The signal of Tau in the medium revealed by the anti-Tau antibody A0024 was less intense than the signal detected after rTau was added to the medium. The two membranes were revealed side by side.
