# Supplementary figures and images for: Unconventional secretion of tau by VAMP8 impacts its intra- and extracellular cleavage
Source: Front Cell Dev Biol. 2022 Oct 5;10:912118. doi: 10.3389/fcell.2022.912118 (PMC9605769; doi:10.3389/fcell.2022.912118)

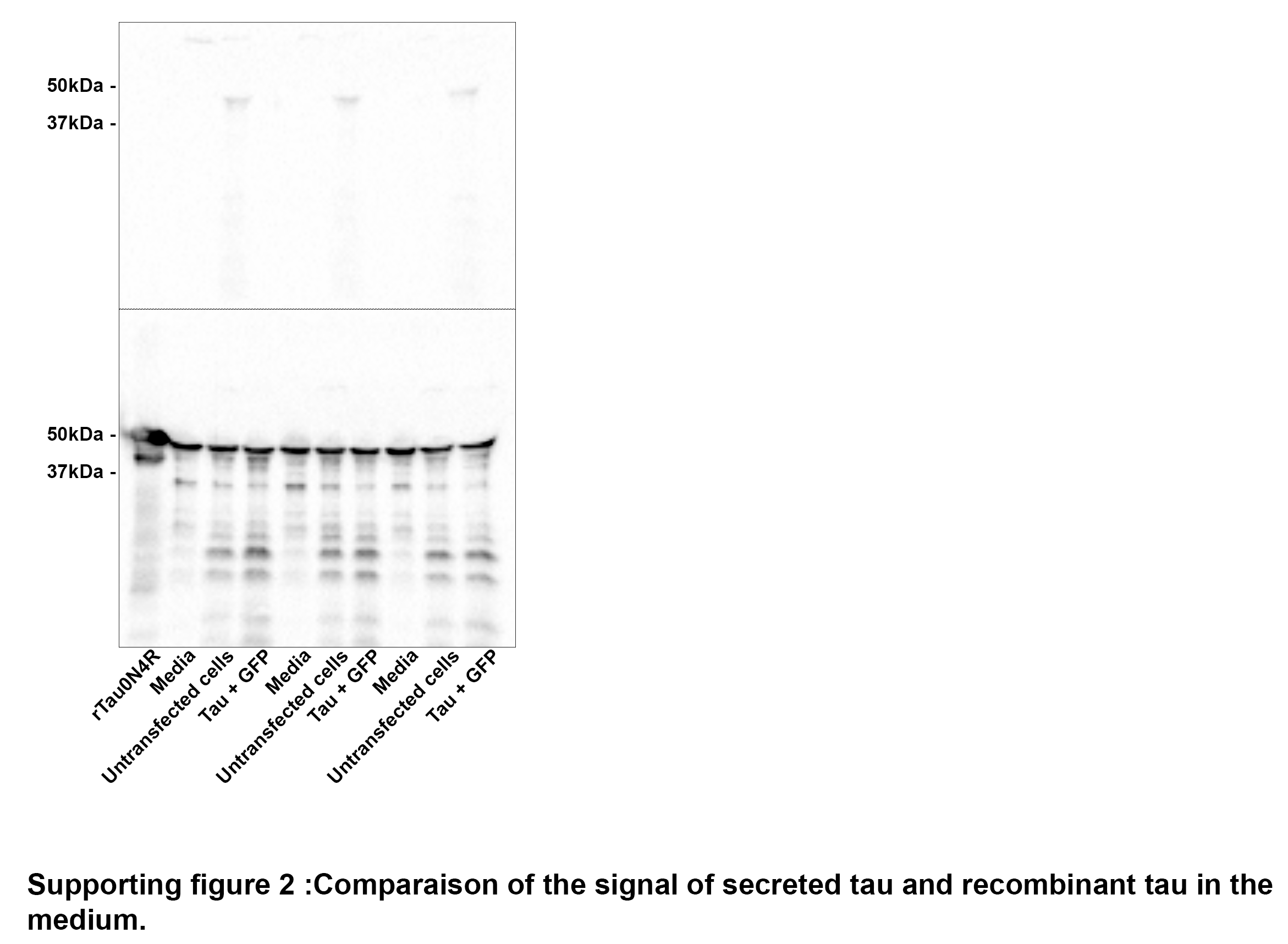

Supplement: Supplementary file 1 [file Image2.tif]

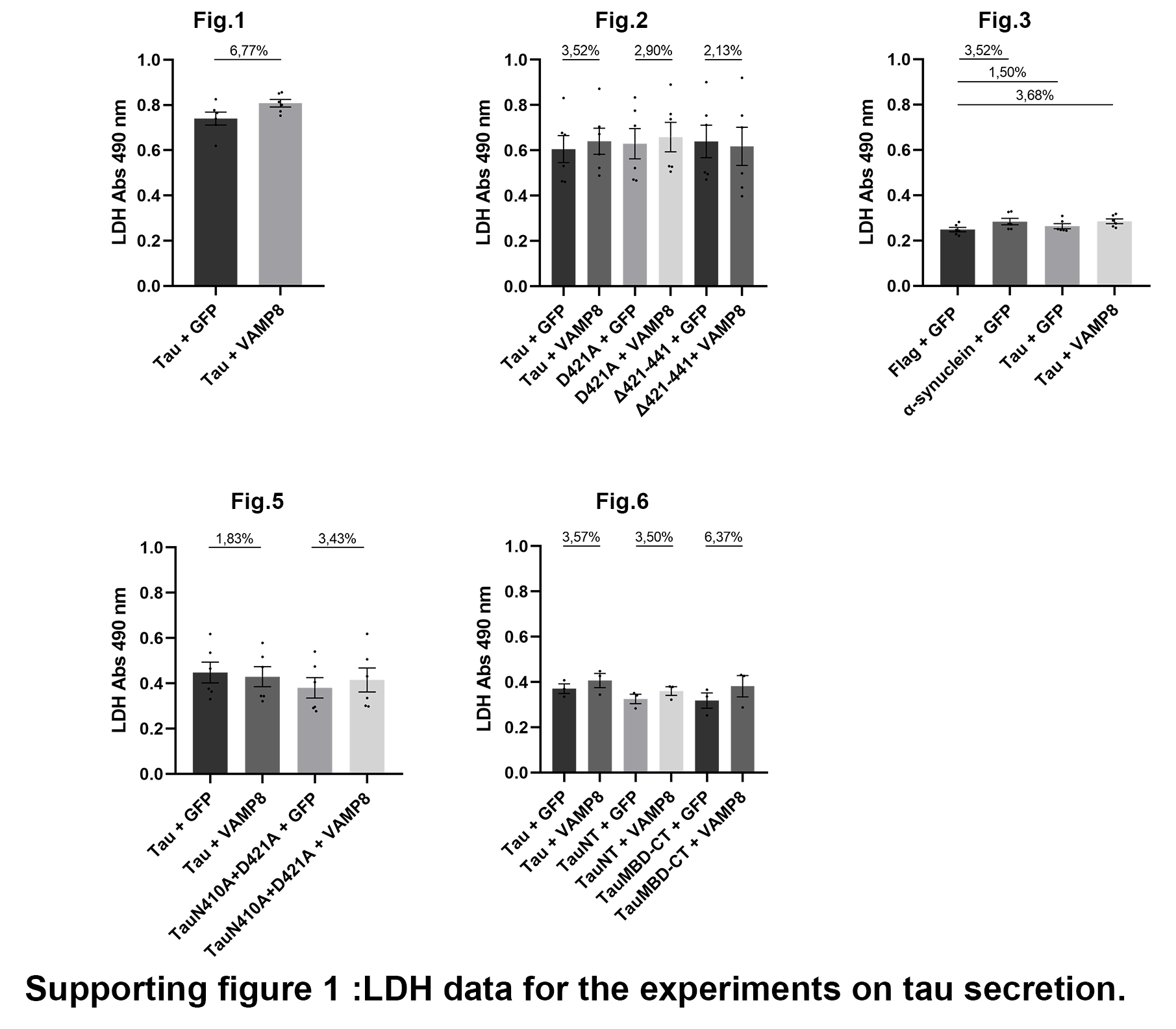

Supplement: Supplementary file 2 [file Image1.tif]
